# Cost-effectiveness of the SEN-concept: Specialized Emergency Nurses (SEN) treating ankle/foot injuries

**DOI:** 10.1186/1471-2474-8-99

**Published:** 2007-10-01

**Authors:** Robert J Derksen, Veerle MH Coupé, Maurits W van Tulder, Bart Veenings, Fred C Bakker

**Affiliations:** 1 Department of Surgery/Traumatology, VU University Medical Center, Amsterdam, The Netherlands; 2 Department of Clinical Epidemiology and Biostatistics, VU University Medical Center Amsterdam, The Netherlands; 3 Institute for Research in Extramural Medicine (EMGO), VU University Medical Center Amsterdam, The Netherlands; 4 Institute for Health Sciences, Faculty of Earth and Life Sciences, VU University, Amsterdam, The Netherlands

## Abstract

**Background:**

Emergency Departments (EDs) are confronted with progressive overcrowding. As a consequence, the workload for ED physicians increases and waiting times go up with the risk of unnecessary complications and patient dissatisfaction. To cope with these problems, Specialized Emergency Nurses (SENs), regular ED-nurses receiving a short, injury-specific course, were trained to assess and treat minor injuries according to a specific protocol.

**Methods:**

An economic evaluation was conducted alongside a randomized controlled trial comparing House Officers (HOs) and SENs in their assessment of ankle and foot injuries. Cost prices were established for all parts of healthcare utilization involved. Total costs of health care utilization were computed per patient in both groups. Cost-effectiveness was investigated by comparing the difference in total cost between groups with the difference in sensitivity and specificity between groups in diagnosing fractures and severe sprains. Finally, cost-effectiveness ratios were calculated and presented on a cost-effectiveness plane.

**Results:**

No significant differences were seen between treatment groups for any of the health care resources assessed. However, the waiting times for both first assessment by a treatment officer and time spent waiting between hearing the diagnosis and final treatment were significantly longer in the HO group. There was no statistically significant difference in costs between groups. The total costs were € 186 (SD € 623) for patients in the SEN group and € 153 (SD € 529) for patients in the HO group. The difference in total costs was € 33 (95% CI: – € 84 to € 155). The incremental cost-effectiveness ratio was € 27 for a reduction of one missed diagnosis and € 18 for a reduction of one false negative.

**Conclusion:**

Considering the benefits of the SEN-concept in terms of decreased workload for the ED physicians, increased patient satisfaction and decreased waiting times, SENs appear to be a useful solution to the problem of ED crowding.

## Background

Emergency Departments (EDs) worldwide are confronted with overcrowding due to physician shortage and a steady growth of patients visiting the ED [1,2]. Long waiting times in the ED lead to unsatisfied patients. Moreover, crowding causes late diagnoses with the possibility of unnecessary complications. To cope with these problems, Advanced Nurse Practitioners (ANPs) and Physician Assistants (PAs) were introduced to take over certain diagnostic tasks from doctors to alleviate the physicians of some of the workload in the ED [3]. These so-called midlevel practitioners have proven to be of good value in the everyday practice of the ED by treating certain injuries and conditions while maintaining a good standard of care and patient satisfaction [4-6].

However, there are also some disadvantages to the development: training is relatively long and expensive and trainees are withdrawn from the already tight nurses pool [7,8]. To resolve these disadvantages and maintain the benefits of these practitioners, we conceived a new concept: the Specialized Emergency Nurse (SEN). Regular emergency nurses received an injury-specific course to assess and treat minor injuries themselves according to a protocol. SENs are trained to be flexible employees capable of treating common injuries alongside their regular nursing duties.

The first injury to test this method on was the ankle sprain, because it is a common injury for which validated clinical decision rules exist that could easily be implemented in an algorithm (Ottawa Ankle and Foot Rules) [9-13].

Before this study, the ability of SENs to clinically assess ankle and foot injuries was compared with junior emergency physicians (house officers [HOs]) in an interobserver agreement study [14]. Furthermore, the accuracy of SENs in interpreting the accompanying radiographs was studied [15]. The results of both studies were promising to such a degree that a randomized controlled trial, the SEN-trial, was set up to assess the ability and consequences of SENs diagnosing and treating patients with an ankle or foot injury compared with HOs. Outcome measures considered in the trial were accuracy of assessment, patient satisfaction, and waiting time. The clinical part of the study revealed comparable accuracy results between the SENs and HOs. The SEN group scored significantly better than the HO group on patient satisfaction and waiting times. These results are presented in a separate paper [16].

At present, no literature is available about the cost-effectiveness of deploying regular nurses to perform diagnostic and treatment tasks after a short, injury-specific course. ANPs and PAs, however, have been proven to be more expensive than HOs [17]. The results of the economic evaluation that was performed alongside the SEN trial are presented in the current paper.

## Methods

### Study design

An economic evaluation was conducted alongside a prospective randomized controlled trial (SEN-trial) comparing House Officers (HOs) and Specialized Emergency Nurses (SENs) in their assessment of ankle and foot injuries in the ED. Assessment was performed by both groups according to a protocol based on the Ottawa Ankle and Foot rules. Effectiveness and cost-effectiveness were investigated. The protocol was approved by the scientific committee and medical ethics committee of the VU University Medical Center and was carried out in accordance with the Declaration of Helsinki (1989) of the World Medical Association.

### Observer groups

Out of 32 certified emergency nurses, 16 volunteered to participate in this trial. The mean age of the nurses was 36 years (range, 26–56 years). The mean clinical experience in the ED for the nurses was 5 years (range, 6 months–12 years).

Before the study started, the nurses were trained in the anatomy and biomechanics (trauma mechanisms) of the ankle and foot, and were taught how to interpret the accompanying radiographs for the detection of acute fractures in a 2-day course, provided by a surgeon and a radiologist. Successful completion of the course led to the SEN qualification. Furthermore, all HOs, 24 in total, participated in the study. The HOs' mean age was 28 years (range, 26–30 years) and their mean clinical experience (in an ED) was 1 year (range, 6 months–1.5 years).

### Study population

Patients were randomly assigned to 1 of the 2 treatment groups by computer, which allocated patients unstratified into the 2 observer groups in blocks of 20. All consecutive patients who came to the ED with an ankle or foot injury were invited to enter the study. Exclusion criteria were: age younger than 18 years or older than 65 years; trauma sustained more than 48 hours before presentation; mental or physical conditions known to complicate assessment of the injury; ankle/foot injuries as part of a more severe (poly)trauma; and prior injury to the ipsilateral ankle/foot that required surgery. Written informed consent was obtained from all patients. For more details on the trial, we refer to the clinical paper [16].

### Clinical outcome measures

The included patients visited the outpatient clinic again after one week. One surgeon reassessed all patients to establish the definitive diagnosis (gold standard). The primary outcome measures were the accuracy parameters calculated as the sensitivity and specificity of both observer groups. These parameters were found by comparing the results of both observer groups to the gold standard (surgeon that reviewed the radiographs and reassessed ankle stability). The secondary outcome measure was patient satisfaction. The clinical outcome measures and their assessment are extensively described in the clinical paper [16].

### Assessment of resource utilization

Data regarding costs directly related to the treatment of ankle and foot injuries were collected for each patient in the trial. The following data were taken into account: number of X-rays performed; use of crutches and duration of use; initial treatment (cast, pressure bandage or tubigrip); admittance to the hospital and duration of hospitalization; operation together with the used materials and duration of operation. Most cost parameters were collected at initial presentation at the ED. Few cost parameters (crutch use and hospitalization elsewhere) were collected at the control visit or by phone (for patients who did not return for the control visit). Data on crutch use were collected for the last 115 patients of the study. These data were used to estimate group means that were consequently imputed for the entire group. Other costs within the health care sector consisted of those associated with setting up the 2-day training course as well as providing it. Furthermore, the costs associated with the time SENs and HOs spent on patients in both groups were included. This was done by prospectively measuring the time spent on clinically assessing the patient as well as the time spent on interpreting the accompanying radiograph (if made). For practical reasons, these data were collected for a selection of patients and imputed for both groups.

The time loss patients experienced during the ED visit (indirect cost of productivity loss) was also taken into account. Data were prospectively collected concerning the time spent in the waiting room of the ED; time between clinical assessment and radiograph (if made), and time spent waiting between hearing the diagnosis and final treatment. The time spent in the waiting room was collected for all patients. The waiting time between clinical assessment and radiograph, and time spent waiting between hearing the diagnosis and final treatment in the ED were, because of practical reasons, collected in a random selection of patients (n = 18). The mean of these data were consequently imputed per group for the rest of the study population (Table 1).

**Table 1:** Mean (SD) invested time spans per treatment group

	SEN			HO		
	*mean*	*(SD)*	*N*	*mean*	*(SD)*	*N*
**Invested time per observer (SEN/HO)**						
Duration clinical assessment (min)	5	(1.3)	*11*	5	(2.2)	*7*
Duration radiograph interpretation (min)	2	(0.8)	*10*	1.8	(1.1)	*5*
Duration review radiograph (min)	0.5	---	*3*	0.5	---	*3*
**Invested time per patient**						
Waiting time 1^st ^assessment – radiograph (min)	37	(12)	*10*	33	(8)	*5*
Waiting time diagnosis – treatment (min)	3	(4)	*11*	16	(14)	*7*

### Valuation of health care consumption; unit costs

The economic evaluation was conducted from a societal perspective. The study was carried out from August 2004 to March 2005. Therefore, 2004 prices were used. For the most relevant cost items, cost prices for the VU University Medical Center were calculated. These cost prices reflect costs of real resource use and include overhead costs. Items with a negligible influence (because of low average numbers) were valued according to the Handbook for economic evaluation in the Netherlands [18]. Table 2 shows the unit cost prices that were used. Waiting time was valued using shadow prices that value time spent at unpaid work or informal care. This shadow price equals the tariff for hiring someone, which was € 8.30 an hour.

**Table 2:** Costs per unit health care resource used in the economic evaluation of SEN (year 2004)

Healthcare resource [Unit]	Cost per unit (€)
Radiograph	42
Crutch-rent per week	4
Tubigrip	7
Pressure bandage	24
Lower extremity cast	68
Hospitalization per day	482
Operating room per hour	1274
Osteosynthesis materials (mean)	270
Hourly fee SEN	30
Hourly fee HO	33
Hourly fee specialist	148
Waiting time patient	8.40*
Training SEN (per SEN)	170

* Shadowprice per hour.

### Statistical analysis

Statistical analysis was carried out according to the intention-to-treat principle. A total of 512 patients were included in the trial: 263 were randomized to the SEN group and 249 to the HO group. The total costs were computed by multiplying resource data by cost prices. The difference between total costs in the SEN group and the HO group were consequently computed and the 95% confidence interval (95% CI) for this difference was calculated. As cost data are typically skewed, confidence intervals for cost differences cannot be estimated with conventional methods that assume normality. To avoid distributional assumptions, we applied the non-parametric bootstrap [19,20]. Basically, in the non-parametric bootstrap, samples of the same size as the original dataset are drawn by sampling with replacement from the observed data. These bootstrap samples can be used to estimate standard errors and confidence intervals. To obtain 95% confidence intervals for cost differences, we performed a non-parametric bootstrap with a 1000 replications [21].

For the cost-effectiveness analysis, the difference in total cost between the two treatment groups was compared with the difference in sensitivity and specificity. In other words, the increase (or decrease) in total costs per missed diagnosis (false positives and negatives) for the SEN group versus the HO group was calculated and constitutes the cost-effectiveness ratio.

Uncertainty around the cost-effectiveness ratios was estimated using the bias corrected and accelerated bootstrapping method (1000 replications) and presented on a cost-effectiveness plane [21,22].

## Results

### Clinical outcomes

SENs are capable of assessing and treating patients with an ankle or foot trauma at least as accurate as HOs. The percentage of false positives and false negatives in the SEN group was 7.4% compared with 8.6% in the HO group. For false negatives alone, these percentages were 2.9% and 4.7%, respectively. These differences were not statistically significant. Moreover, SENs accomplished these results with better patient satisfaction results than did the HOs. Furthermore, waiting times were decreased for patients with this type of injury. Full details on the clinical outcomes are presented in a separate paper [16].

### Resource use

Table 1 summarizes the time measurements. No significant differences were found between treatment groups with regard to the invested time of either SEN or HO. Also, as expected, the waiting time for patients between clinical assessment and radiograph (if made) were not different between groups. This waiting period depends on the pressure of activities at the radiology department and is in no way associated with the observer group. Finally, the time spent in the waiting room of the ED and the time spent between hearing the diagnosis and receiving final treatment (eg, application of pressure bandage) was significantly longer in the HO group.

Table 3 lists the mean utilization of health care resources for both treatment groups. No significant differences were seen between the two treatment groups for any of the health care resources measured (X-ray indication; use of crutches and duration of use; treatment modality (cast, pressure bandage or tubigrip); hospital admittance/duration of hospitalization; and duration of operation. However, the waiting time for first assessment by a treatment officer was significantly longer in the HO group.

**Table 3:** Mean (SD) healthcare utilisation per treatment group

	SEN (n = 261)			HO (n = 249)		
	*mean*	*(SD)*	N*	*mean*	*(SD)*	N*
Health care Resource						
Radiograph	83%	(38%)		76%	(43%)	
Crutch-use	67%	(47%)	*45*	67%	(47%)	*70*
Duration of Crutch-use (days)	9.2	(8.2)	*21*	8.2	(5.0)	*28*
Tubigrip	32%	(47%)		36%	(48%)	
Pressure bandage	38%	(49%)		41%	(49%)	
Lower extremity cast	22%	(42%)		19%	(40%)	
Number of hospital admittances	3%	(17%)		2%	(15%)	
Duration of hospitalization (days)	3.3	(2.9)	*7*	2.8	(1.5)	*5*
Duration of operation (min)	83	(35)	*7*	95	(22)	*5*
Invested time patient						
Time spent in waiting room (min)	27	(23)	*200*	41	(35)	*231*

* Number of patients if different from heading.

### Costs

Table 4 shows the mean (standard deviation) costs for the two treatment groups. Direct health care costs were not significantly different between the two groups. These included the costs associated with educating the SENs. Costs outside the health care sector were also not significantly different between groups. As such, the total direct costs were similar in both treatment groups. A substantial part of the direct health care costs was attributable to hospitalization and operation. Note that the indication for hospitalization and operation is set by a supervising surgeon and is, therefore, independent of the treatment group. Furthermore, the mean time spent in the waiting room was significantly shorter in the SEN group compared with the HO group. However, in absolute values, these costs were very small in comparison to the total costs. The total costs were € 186 (SD € 623) per patient in the SEN group and € 153 (SD € 529) per patient in the HO group. The difference in total costs was € 33 (95% CI: – € 84 to € 155). This difference could be explained by the difference in hospital admittance/operation and training costs for the SENs, and was not statistically significant.

**Table 4:** Mean (SD) costs for treatment group for all patients

	SEN (*n *= 262)		HO (*n *= 249)		**Difference (95% CI)**^**†**^
	*mean*	*(SD)*	*mean*	*(SD)*	
***HEALTH CARE SECTOR***					

Resource use					
Radiograph	34.5	(15.7)	31.8	(17.8)	2.7 (-0.3 ; 5.9)
Crutch-use	4.7	(5.8)	4.4	(4.2)	0.3 (-0.5 ; 1.2)
Tubigrip	2.3	(3.3)	2.5	(3.4)	-0.3 (-0.9; 0.4)
Pressure bandage	9.4	(26.6)	10.0	(26.9)	-0.6 (-3 ; 2)
Lower extremity cast	15.2	(28.5)	13.2	(27.1)	2.0 (-2.3; 6.3)
Costs related to operation					
Hospitalization	42.3	(333)	27.1	(210)	15.2 (-33; 65)
OR/osteosynthesis materials	52.9	(342)	46.9	(333)	6.0 (-69 ; 81)
Valuation of time of SEN or HO					
Clinical assessment	2.5	---	2.8	---	-0.2 *
Radiographic assessment	0.8	---	0.8	---	0.1 *
Review radiograph	2.5	---	2.3	---	0.2 *
Training SEN	10.4	---	0	---	10.4 *

***INDIRECT HEALTH CARE COSTS***					

Time spent in waiting room	3.8	(3.3)	5.7	(4.7)	-1.9 (-2.6 ; -1,2)
Waiting time assessment – radiograph	4.0	---	3.7	---	0.3 *
Waiting time diagnosis – treatment	0.4	---	2.2	---	-1.8*

***Total health care costs***	**178**	**(622)**	**142**	**(529)**	**36 (-50 ; 123)**
***Total indirect health care costs***	**8**	**(4)**	**12**	**(5)**	**-3 (-4 ; -2)**
***Total costs***	**186**	**(623)**	**153**	**(529)**	**33 (-84 ; 155)**

^† ^CI estimated with a non-parametric bootstrap with 1000 replications

* CI not available; estimate at group level

### Cost-effectiveness

Table 5 shows the total costs and effects. Table 6 displays the differences in total costs and the differences in effect together with the incremental cost-effectiveness ratios. The effect differences were 1.2% for the false negatives and positives combined and 1.8% for the false negatives alone. Considering the cost-difference between treatment groups of € 33, the incremental cost-effectiveness ratio for reduction of false negatives and positives was € 27 and the incremental cost-effectiveness ratio for reduction of false negatives alone was € 18. In other words, SEN cost an additional € 27 per false positive or negative that is avoided. Furthermore, SEN cost an additional € 18 per false negative (missed fracture/total collateral ligament rupture) that is avoided.

**Table 5:** Mean costs and effects by treatment group for all patients (missing data imputed)

	SEN (n = 242)		HO (n = 233)	
	
*Effect measure*	*Costs*	*Effects*	*Costs*	*Effects*
False positives and false negatives	186	7.4 %	153	8.6 %
False negatives	186	2.9 %	153	4.7 %

**Table 6:** Mean cost and effect differences between treatment groups and cost-effectiveness ratios for all patients (missing data imputed)

	SEN- HO		
*Effect measure*	*Cost difference*^†^	*Effect difference*^†^	*Incremental Cost-effectiveness ratio*
Decrease in false positives and false negatives	33	1.2 %	27
Decrease in false negatives	33	1.8 %	18

**Figure 1:**
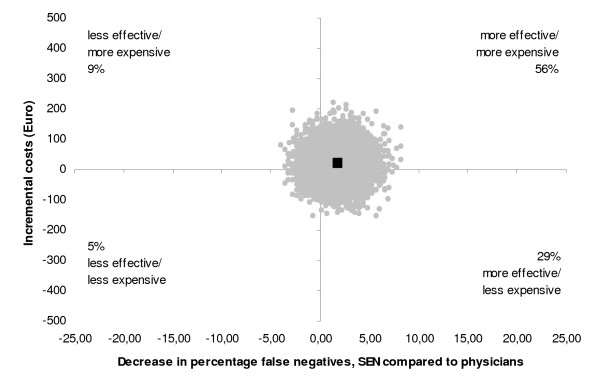
**Cost-effectiveness plane**. Cost-effect pairs are displayed as dots (coordinates) in the grid. On the Y-axis, incremental costs are displayed; on the X-axis, the difference in sensitivity is displayed between SENs and physicians.

Figure 1 shows the cost-effectiveness plane for true positives gained. 65 percent of the cost-effect pairs lie above the x-axis, the area where SEN deployment is associated with higher costs. Furthermore, 85 percent of cost-effect pairs lie right to the y-axis, the area where SEN deployment is associated with more effect. Note that the differences are small and the spreading is close to zero.

## Discussion

Differences in total costs between the SEN group and HO group were not statistically significant. In daily practice, this means that we seem to have found an attainable solution to ED crowding that reduces waiting times, increases patient satisfaction and reduces workload for emergency physicians without increasing the costs. SEN cost only € 27 per false positive or negative that is avoided. The small difference in total costs in favour of the HO group is largely attributable to the slight (non-significant) difference in number of hospital admittances/operations, that accounts for larger costs in the SEN group. These costs are random since hospital admittance/operation is not a decision made by the observer, though is made by a supervising surgeon. Furthermore, the cost difference could partly be attributed to the costs of educating SENs. These costs are overestimated, since most of these costs were made for developing the educational programme and are not needed when a second group of nurses is trained. In addition, once a nurse has received training, the more patients he or she treats, the lower are the additional costs per patient.

Only few earlier publications on the costs and cost-effectiveness of alternative solutions to the problem of ED crowding are available. In the case of ANPs/PAs, one important publication revealed that ANP/PA delivered care was associated with higher costs than the standard care provided [17]. In light of these findings by Sakr et al., it seems that the SEN concept might constitute an attractive alternative to the currently available midlevel practitioners. SENs provide the benefits of deploying nurses to do a physicians' job as do ANPs/PAs: shorter waiting periods, decreased workload for emergency physicians and increased patient satisfaction while maintaining excellent diagnostic accuracy [16]. However, the costs associated with deploying SENs seem to be lower than those associated with the deployment of ANPs/PAs. The latter are more expensive because of the higher wages and costly educational programmes involved. Furthermore, it is important to note that the same results would have been seen when HOs/Emergency Physicians would be more expensive. This is due to the fact that the costs associated with income of the observer constitute only a small part of the total costs and therefore are irrelevant to the overall costs and consequently the cost difference.

### Strengths and limitations of the study

For a critical appraisal of the design of the study, we refer to the clinical paper [16]. With respect to the collection of information on utilization of some resources and (waiting/assessment) times, some limitations should be mentioned. Most resources were collected for all patients. However, as for crutch use, these data were collected for the last 115 patients and imputed for the rest of the groups. With regard to invested times by patients, the overall time spent in the ED as well as time spent in the waiting room were collected for all patients. However, waiting times between hearing the diagnosis and final treatment were collected for a limited number (N = 18) of patients per group and then imputed for both observer groups. As for the invested assessment times by the observers, these times were all recorded in a limited number of patients (N = 18) and then imputed for both observer groups. When imputing the mean values from the mentioned data, the variance was ignored. Obviously, it would have been better if we had collected these data for all patients. However, the sensitivity analysis performed later, resulted in similar results and therefore, the method used seems justified. Furthermore, since the effect is an underestimation of the variance, an increase in the variance will only affirm the observation that cost differences between the groups are not significant and therefore will not influence the conclusions drawn.

For the remaining resource variables, very few data were missing and considering the large sample size, estimates are considered to be accurate. With over 500 patients being included, the study is well-powered. Finally, all data were collected prospectively, contributing to the strength with which conclusions can be drawn from the presented results.

## Conclusion

Considering the results of this study and keeping in mind the results of the accompanying clinical trial, costs and effects are equal for both the SEN group and the HO group. SENs cost only € 27 per false positive or negative and only € 18 per false negative that is avoided. Considering the benefits of the SEN-concept in terms of decreased workload for the physicians working in the ED, the increased patient satisfaction and shorter waiting times in the SEN group, SENs appear to be an attractive solution to the problem of ED crowding.

## Competing interests

The author(s) declare that they have no competing interests.

## Authors' contributions

RJD carried out the principle trial and the accompanying cost-effectiveness analysis, he designed the study and drafted the manuscript. VMHC co-designed the cost-effectiveness trial and performed the statistical analysis. MWT participated in the design of the study. BV participated in the coordination of the study. FCB supervised and co-designed the study. All authors read and approved the final manuscript.

## Pre-publication history

The pre-publication history for this paper can be accessed here:

http://www.biomedcentral.com/1471-2474/8/99/prepub

## References

[B1] Van Geloven AA Luitse JS Simons MP Volker BS Verbeek MJ Obertop H Emergency medicine in the Netherlands, the necessity for changing the system: results from two questionnaires Eur J Emerg Med 2003 10 318 322 10.1097/00063110-200312000-00015 14676512

[B2] Singh S Self referral to accident and emergency department: patients' perceptions BMJ 1988 297 1179 1180 3144340 10.1136/bmj.297.6657.1179 PMC1835022

[B3] Dealey C Emergency nurse practitioners: should the role be developed? Br J Nurs 2001 10 1458 1468 11842461 10.12968/bjon.2001.10.22.9341

[B4] Sakr M Angus J Perrin J Nixon C Nicholl J Wardrope J Care of minor injuries by emergency nurse practitioners or junior doctors: a randomised controlled trial Lancet 1999 354 1321 1326 10.1016/S0140-6736(99)02447-2 10533859

[B5] Ganapathy S Zwemer FL Jr. Coping with a crowded ED: an expanded unique role for midlevel providers Am J Emerg Med 2003 21 125 128 10.1053/ajem.2003.50030 12671813

[B6] Cooper MA Lindsay GM Kinn S Swann IJ Evaluating Emergency Nurse Practitioner services: a randomized controlled trial J Adv Nurs 2002 40 721 730 10.1046/j.1365-2648.2002.02431.x 12473052

[B7] Murray MK The nursing shortage. Past, present, and future J Nurs Adm 2002 32 79 84 10.1097/00005110-200202000-00005 11984233

[B8] Mandrell BN Hobbie W Deatrick J Lipman T Hinds PS When recruiting advance practice nurses is costly, develop your staff nurses J Nurs Adm 2004 34 543 545 15632748 10.1097/00005110-200412000-00002

[B9] Stiell IG Greenberg GH McKnight RD Nair RC McDowell I Worthington JR A study to develop clinical decision rules for the use of radiography in acute ankle injuries Ann Emerg Med 1992 21 384 390 10.1016/S0196-0644(05)82656-3 1554175

[B10] Stiell IG Greenberg GH McKnight RD Nair RC McDowell I Reardon M Stewart JP Maloney J Decision rules for the use of radiography in acute ankle injuries. Refinement and prospective validation JAMA 1993 269 1127 1132 10.1001/jama.269.9.1127 8433468

[B11] Pigman EC Klug RK Sanford S Jolly BT Evaluation of the Ottawa clinical decision rules for the use of radiography in acute ankle and midfoot injuries in the emergency department: an independent site assessment Ann Emerg Med 1994 24 41 45 7912053 10.1016/s0196-0644(94)70160-1

[B12] Auleley GR Kerboull L Durieux P Cosquer M Courpied JP Ravaud P Validation of the Ottawa ankle rules in France: a study in the surgical emergency department of a teaching hospital Ann Emerg Med 1998 32 14 18 10.1016/S0196-0644(98)70093-9 9656943

[B13] Bachmann LM Kolb E Koller MT Steurer J ter Riet G Accuracy of Ottawa ankle rules to exclude fractures of the ankle and mid-foot: systematic review BMJ 2003 326 417 12595378 10.1136/bmj.326.7386.417 PMC149439

[B14] Derksen RJ Bakker FC Geervliet PC ES LK Heilbron EA Veenings B Patka P Haarman HJ Diagnostic accuracy and reproducibility in the interpretation of Ottawa ankle and foot rules by specialized emergency nurses Am J Emerg Med 2005 23 725 729 10.1016/j.ajem.2005.02.054 16182978

[B15] Derksen RJ Bakker FC Heilbron EA Geervliet PC Spaans IM ES LK Veenings B Patka P Haarman HJ Diagnostic accuracy of lower extremity X-ray interpretation by 'specialized' emergency nurses Eur J Emerg Med 2006 13 3 8 10.1097/00063110-200602000-00002 16374240

[B16] Derksen RJ Bakker FC ES LK Spaans IM Heilbron EA Veenings B Haarman HJ Specialized emergency nurses treating ankle and foot injuries: a randomized controlled trial Am J Emerg Med 2007 25 144 151 10.1016/j.ajem.2006.06.011 17276802

[B17] Sakr M Kendall R Angus J Sanders A Nicholl J Wardrope J Saunders A Emergency nurse practitioners: a three part study in clinical and cost effectiveness Emerg Med J 2003 20 158 163 10.1136/emj.20.2.158 12642530 PMC1726060

[B18] Oostenbrink JB Koopmanschap MA Rutten FF Standardisation of costs: the Dutch Manual for Costing in economic evaluations Pharmacoeconomics 2002 20 443 454 10.2165/00019053-200220070-00002 12093300

[B19] Barber JA Thompson SG Analysis of cost data in randomized trials: an application of the non-parametric bootstrap Stat Med 2000 19 3219 3236 10.1002/1097-0258(20001215)19:23<3219::AID-SIM623>3.0.CO;2-P 11113956

[B20] Thompson SG Barber JA How should cost data in pragmatic randomised trials be analysed? BMJ 2000 320 1197 1200 10784550 10.1136/bmj.320.7243.1197 PMC1127588

[B21] Briggs AH Wonderling DE Mooney CZ Pulling cost-effectiveness analysis up by its bootstraps: a non-parametric approach to confidence interval estimation Health Econ 1997 6 327 340 10.1002/(SICI)1099-1050(199707)6:4<327::AID-HEC282>3.0.CO;2-W 9285227

[B22] Briggs A Fenn P Confidence intervals or surfaces? Uncertainty on the cost-effectiveness plane Health Econ 1998 7 723 740 10.1002/(SICI)1099-1050(199812)7:8<723::AID-HEC392>3.0.CO;2-O 9890333

